# Acute exercise-induced enhancement of fear inhibition is moderated by BDNF Val66Met polymorphism

**DOI:** 10.1038/s41398-019-0464-z

**Published:** 2019-04-09

**Authors:** Dharani Keyan, Richard A. Bryant

**Affiliations:** University of New South Wales, Sydney, Australia

## Abstract

Rodent research indicates that acute physical exercise facilitates fear learning and inhibition. Expression of brain-derived neurotrophic factor (BDNF) may moderate the memory enhancing effects of acute exercise. We assessed the role of acute exercise in modulating extinction retention in humans, and investigated the extent to which the BDNF polymorphism influenced extinction retention. Seventy non-clinical participants engaged in a differential fear potentiated startle paradigm involving conditioning and extinction followed by random assignment to either intense exercise (*n* = 35) or no exercise (*n* = 35). Extinction retention was assessed 24 h later. Saliva samples were collected to index BDNF genotype. Exercised participants displayed significantly lower fear 24 h later relative to non-exercised participants. Moderation analyses indicated that after controlling for gender, the BDNF Val66Met polymorphism moderated the relationship between exercise and fear recovery 24 h later, such that exercise was associated with greater fear recovery in individuals with the Met allele. These findings provide initial evidence that acute exercise can impact fear extinction in humans and this effect is reduced in Met-allele carriers. This finding accords with the role of BDNF in extinction learning, and has implications for augmenting exposure-based therapies for anxiety disorders.

## Introduction

Fear extinction is a form of inhibitory associative learning whereby a previously conditioned stimulus (CS) becomes associated with safety via repeated exposure to it in the absence of an aversive outcome^1^. This new safety memory competes with the original fear conditioned memory, such that under certain conditions it can function to actively inhibit fear expression^2^. Fear extinction forms the basis of exposure therapy for anxiety-related disorders. Despite strong empirical validation for exposure therapy^3,4^, many individuals do not optimally benefit from this treatment and this has prompted an exploration of ways to enhance retention of fear extinction.

Consolidation of fear extinction and its retrieval relies upon modifications in crucial brain networks responsible for fear extinction. Central to extinction is brain-derived neurotrophic factor (BDNF), a neurotrophin important for regulating synaptic plasticity^5^ that is secreted in an activity-dependant manner^6^ via preferential binding to the receptor tropomyosin receptor kinase B (TrkB^7^), and has been shown to modulate fear recovery. Animals that fail to learn extinction display reduced BDNF in hippocampal afferents to the infralimbic-PFC, and augmenting BDNF in these networks restores this learning deficit^8^. Further, inhibition of Trk-B signalling within the basolateral amygdala prior to extinction training leads to deficits in extinction retention but not within session extinction, which implicates BDNF in the consolidation of extinction as well^9^. A genetic variant involving a substitution from valine (Val) to methionine (Met) at codon 66 within the pro region (Val66Met) has been associated with lower-activity-dependent BDNF secretion in brain regions critical for fear extinction^10^. Relatedly, genetically modified mice with this variant that have partial reductions in BDNF secretion display impaired contextual fear conditioning, with infusions of BDNF partially rescuing these memory deficits^11^. In humans, the Val66Met polymorphism has been associated with enhanced amygdala activity during extinction^12^ and impaired subsequent extinction learning that appears related to reduced activation of the vmPFC region^13^. Further, BDNF signalling appears to interact with glucocorticoid production^14^ within the context of long-term memory as well, with consolidation of glucocorticoid-mediated inhibitory learning necessarily relying upon selective recruitment of BDNF pathways in the hippocampus^15^. Underscoring the relevance of BDNF to extinction retention, there is evidence that carriers of the BDNF-Met allele (characterised by depleted expression of BDNF) are less likely to respond to exposure therapy than Val allele carriers^16^.

There is growing evidence for the role of brief physical exercise in modulating learning and memory in animals^17–22^ and humans^23–25^. Animal studies evidence upregulation of exercise-induced BDNF in critical brain regions including the hippocampus^26^, amygdala^27^ and prefrontal cortex^28^. In humans, BDNF levels are upregulated following acute bouts of exercise^25^, with some studies evidencing a dose-dependent relationship such that higher intensities are associated with elevated levels of BDNF^29,30^. Apart from BDNF, the noradrenergic and glucocorticoid systems implicated in fear extinction, are also involved in the beneficial effects of exercise. Specifically, blocking the noradrenergic system dampens BDNF mRNA expression in exercised rats^31,32^, suggesting an intact noradrenergic system as critical for hippocampal BDNF mRNA transmission. Furthermore, voluntary wheel-running in animals activates the hypothalamic–pituitary–adrenal (HPA) axis and related glucocorticoid receptors^33–35^, and facilitates exercise-induced memory formation in animals^20^. Thus, it appears that exercise-induced release of BDNF and stress hormones may operate in concert when modulating subsequent retention of fear extinction process.

Recent animal work has demonstrated that a single bout of immediate exercise following extinction training of conditioned fear subsequently reduced expression of fear on a delayed retention test^19,22^. Importantly, a dose-response effect of exercise on extinction memory retention has been evidenced whereby distance covered in the wheel running correlated with reduced fear^22^. To date, no study has extended these findings in humans. In this study, we aimed to examine the effect of an acute bout of exercise in modulating extinction retention. It is hypothesised that intense exercise immediately following extinction training would consolidate this information such that memory tested 24 h later would be better retained than a control condition. Further, individual differences owing to the BDNF Val66Met polymorphism may modulate the beneficial effects of exercise on extinction memory retention. Physical exercise may magnify genotype-dependent differences such that Val homozygotes relative to Met allele carriers would benefit more from exercise, and accordingly displaying enhanced extinction retention. That is, we expected Met allele carriers to display greater fear recovery following exercise relative to Val carriers.

## Methods and materials

### Participants

Ninety-seven healthy individuals aged between 18 and 35 years (*M* = 20.64; SD = 3.18) participated in exchange for either course credit or monetary compensation. Participants’ baseline emotional state was assessed using the Depression Anxiety and Stress Scales (DASS-21^36^) 21-item version. Participants indicating any significant health condition were excluded, such that any illness was not further exacerbated by the exercise task (nil were excluded). Participants’ habitual exercise routine (detailed in the Supplement) was measured using the Godin–Shephard Leisure-Time Exercise Questionnaire (LTEQ^37^). A total of 27 participants were excluded for the following reasons: 12 participants were excluded because they exhibited virtually no eye blinks; 2 were excluded as their unconditioned startle responses (as determined in the habituation and/or preconditioning phases) were at least 3.5 standard deviations above the mean; 1 was excluded due to moderately strenuous activity completed while in the no exercise condition (as indicated by mean HR); 5 participants were excluded as they responded with a ‘moderate’ or greater level of depression, stress or anxiety on the DASS-21; and 7 participants dropped out mid-way and/or following session 1 of the study. The final sample consisted of 70 participants of whom 35 were in the exercise condition (20 male, 15 female), and 35 in the control condition (20 male, 15 female). Participants were from an ethnically heterogeneous population including 38.6% Caucasian (*n* = 27), 51.4% Asian (*n* = 36) and 10% other or mixed race (*n* = 7) origin, and this did not differ by Experimental Condition (*p* > 0.05).

### Materials

#### Exercise task

A validated maximal cycle protocol^38^ was adapted as the exercise intervention to account for individual fitness levels, which has been shown to modulate exercise-induced memory^39^. While prior exercise testing was not utilised to determine participants’ fitness level, a modified protocol incorporated individualised workload during the exercise intervention. Participants were required to engage in 20–25 min of incremental cycling at a cadence between 60 and 70 revolutions per minute (RPM) on a manual cycle ergometer (Monark 828E, Sweden). The task began with a 3-min warm up period, after which the workload was increased by 1/4 kg (i.e. kilopond) per minute until participants reached their limit of tolerance, which was defined as the ability to no longer maintain a cadence of at least 60 RPM (i.e. maximal resistance^38^). Following this time, frictional resistance was reduced just enough to meet the minimum cadence (i.e. 60 RPM; submaximal resistance), which was thereafter maintained until the end of the task. Participants’ exercise performance was closely monitored such that when heart rate (detailed in the Supplement) dropped below a minimum standard (arbitrary rate of 160 bpm), they were notified and accordingly encouraged to increase the intensity. On average, participants took 10 min to reach their maximal resistance level, and thereafter engaged at their submaximal resistance level for at least 10 min of the exercise intervention. It is worth noting that some participants in the exercise condition were unable to maintain a steady state of exercise at their submaximal level for the remainder of the exercise intervention (especially following prior work towards their maximal resistance). As such, these participants were encouraged to engage in interval training via varied cadence (e.g. 30 s at 50 RPM, followed by 1 min at 65 RPM) such that the task could be completed. Participants’ exertion levels were monitored using intermittent RPE ratings (detailed in the Supplement), and were verbally encouraged by the experimenter to provide a true maximal effort (details relating to calculation of participant workload is detailed in the Supplement). The no exercise condition engaged in 20–25 min of easy cycling at a 0-resistance level, and cadence between 60 and 70 RPM, such that factors others than exertion matched the exercise condition.

### Procedure

Following informed consent, participants underwent a 2-day differential fear conditioning and extinction paradigm (detailed in the Supplement) that was adapted from previous human fear-potentiated startle paradigms^40,41^. The conditioned stimuli (CSs) were yellow and purple square geometric shapes, which were placed within a grey or white colour background (contexts; CXs), where one served as the ‘conditioning context’ and the other as the ‘extinction context’ (Supplemental Fig. S1a). The selection of the CSs and CXs were randomly determined and counterbalanced across participants. The unconditioned stimulus (US) was a 0.5 s mild electric shock delivered to the inside of the wrist of the participant, the level of which was predetermined with a threshold test (range, 3–19.8 mA), until participants indicated that the shock was ‘highly annoying but not painful’^42^. Day 1 of the experiment consisted of 4 phases: startle habituation, preconditioning, conditioning and extinction. The *startle habituation phase* consisted of nine startle stimuli that were presented in varied duration ranging from 8 to 12 s to establish a stable baseline level. The *preconditioning phase* assessed the unconditioned effect of the CSs (where the US was not administered) and consisted of 4 CS+ and 4 CS− trials, which were presented within either the to-be conditioned context (CX+), or the to-be extinguished context (CX−). The *conditioning phase* followed after a 2 min break, and consisted of 16 CS+ and 16 CS− trials, all presented within the CX+. The CS+ was reinforced with a shock on 62.5% of the trials; the CS− was never followed with a shock in this phase. The *extinction phase* followed after a 5 min break and consisted of 12 CS+ and 12 CS− trials, all presented within the CX−, and no shocks were delivered in this phase. The extinction phase was designed to have lesser trials relative to the conditioning phase, such that a potential floor effect could be minimised^40^, and an effect of exercise could be detected. Immediately after the conditioning and extinction procedures, participants were randomised to engage in either 20 min of intense cycling (i.e. exercise group), or 20 min of mild cycling (i.e. no exercise), after which the session was terminated. Day 2 of the experiment (24 h after conditioning and extinction training) began with a startle habituation phase similar to Day 1, and this was followed by a *recall phase* consisting of 8 CS+ and 8 CS− trials presented within the CX−, where no shocks were delivered. As the recall phase can be considered additional extinction training sessions, the conditioned response (CR) to the first 2 trials was used as a measure of extinction recall in primary analyses. Each trial began with a 0.5 s fixation cross followed by a 10.5 s context presentation: 3.5 s alone followed by 7.5 or 7.0 s in combination with the CS+ or CS−, respectively (Supplemental Fig. S1b). The mean inter-trial interval (ITI) was 20 s (range: 16–24 s). Online US expectancy ratings were assessed for each trial during the preconditioning, conditioning, extinction, and recall phases (detailed in the Supplement).

### Genetic analyses

Saliva samples were collected using Oragene DNA collection kit (DNA Genotek, Ottawa, Canada). BDNF Val66Met genotypes were determined using iPLEX Gold™ primer extension followed by mass spectrometry analysis on the Agena Bioscience MassARRAY system (Agena Bioscience, San Diego, CA) by the Australian Genome Research Facility [http://www.agrf.org.au/]. The genotype distribution in the current study was 47.1% Val/Val (*n* = 33), 28.6% Val/Met (*n* = 20) and 22.9% Met/Met (*n* = 16). Genotyping frequency analyses were conducted as part of a larger sample consisting of varied studies (data unpublished), and this did not significantly differ from Hardy–Weinberg equilibrium (*χ*^2^ (1) = 3.81, *p* > 0.05). In line with previous research^13,16^, Met allele carriers (Val/Met and Met/Met genotypes) were grouped together due to the rarity of Met/Met genotype. Following this configuration, there were 33 BDNF Val/Val homozygotes, and 36 BDNF Met-66 allele carriers. There was no statistically significant association between gender and BDNF genotype (*χ*^2^ (1, *N* = 69) = 0.029, *p* > 0.05). There was a significant association between ethnicity and BDNF genotype, such that individuals of an Asian origin were more likely to be a carrier of the Met allele than those of a Caucasian origin (*χ*^2^ (2, *N* = 69) = 12.14, *p* = 0.002).

### Primary data analyses

Fear potentiated startle was used as a measure of conditioned fear (detailed in the Supplement). For the habituation phase, a 2 (Experimental Condition) × 3 (Assessment Block) repeated measures ANOVA of startle responses was conducted. For the preconditioning phase, variables were analysed in 2 (Experimental Condition) × 2 (Conditioned Type: CS+, CS−) repeated measures ANOVAs. For the conditioning and extinction phases, variables were analysed in a 2 (Experimental Condition) × 2 (Conditioned Type: CS+, CS−) x 4 (Assessment Block) repeated measures ANOVA. When Mauchly’s test for the assumption of sphericity was violated for the above analyses, the respective Greenhouse–Geisser correction was used. For contingency awareness during session 1, US expectancy ratings of each CS trial was subjected to repeated measures ANOVA, with Trial Number and Conditioned Type as within-subject factors, and between-subjects factor of Experimental Condition. Pre-existing differences in regular physical activity engagement were assessed using a non-parametric Wilcoxon test.

To assess whether BDNF polymorphism moderated the relationship between acute exercise and total fear recovery, a moderation analysis using the PROCESS Macro v. 3 (Model 1) in SPSS v. 25^43^. This moderation analysis was conducted with condition (Exercise vs. Control) as the predictor (X) variable, BDNF Val66Met genotype as the moderator (W) variable, and percent fear recovery. (Averaged FPS to the CS+ across 8 trials in the recall (i.e. retention test) phase divided by the largest FPS to the CS+ in the conditioning phase (multiplied by 100) as the outcome (Y) variable.) Given the importance of BDNF in consolidation of fear conditioning^44–46^, we sought to account for any genotype dependant variation in conditioned fear learning when exploring fear recovery on a retention test 24 h post extinction training. Moderation analysis corrected for heteroscedasticity using the conservative HC4 estimator^47,48^. As the BDNF polymorphism has been found to interact with gender in modulating reactivity to mental^49^ and physical stressors^50,51^, gender was added as a covariate in all genotype dependant analyses.

## Results

### Participant characteristics

Table 1 presents participant characteristics. Planned comparisons revealed no differences between groups in DASS-21 scores, LTEQ scores, age and baseline HR (*p*’s > 0.1).

**Table 1:** Mean participant characteristics

	Exercise (*n* = 35)	No exercise (*n* = 35)	*t*	*p*
	*M* (SD)	*M* (SD)		
Age	21.37 (3.90)	19.91 (2.05)	1.96	0.056
Shock level	4.75 (1.94)	5.76 (3.75)	−1.42	0.162
DASS-Depression	2.46 (2.28)	2.60 (2.63)	−0.24	0.809
DASS-Anxiety	2.97 (2.32)	2.80 (2.47)	0.30	0.766
DASS-Stress	4.09 (2.80)	4.26 (3.45)	−0.23	0.820
LTEQ light intensity (minutes)^a^	30.00 (480.00)	60.00 (420.00)	503.00	0.188
LTEQ moderate intensity (minutes)^a^	60.00 (450.00)	60.00 (900.00)	529.50	0.324
LTEQ strenuous intensity (minutes)^a^	52.50 (375.00)	75.00 (1080.00)	584.00	0.735
Baseline HR	65.68 (11.05)	71.69 (13.03)	−1.48	0.149
Max HR	178.43 (14.65)	86.41 (12.07)*	22.34	<0.001*
Maximal watts	156.93 (39.00)	N/A		
Submaximal watts	129.16 (36.37)	N/A		

Max HR refers to the highest heart rate endured during the experimental manipulation. Median and range (in parentheses) for LTEQ subscales have been presented

*Significant *p* values < 0.05

^a^Non-parametric Mann–Whitney *U* statistic reported

### Heart rate

Raw scores for baseline and maximum HR (HR) are presented in Table 1. Maximum heart rate data was missing for 14 exercised and 16 non-exercised participants due to technical difficulties, and as such were replaced with the mean for the respective group (We conducted an additional analysis of the heart rate data that omitted these 14 exercise and 16 no exercise participants. The results of this analysis do not differ from the results of the analyses that replaced these data with the mean for each respective group. For the restricted sample, *t*-tests revealed that the max *t*(1,38) = 22.34, *p* < 0.001 heart rates differed significantly between groups.) (nil outliers detected as defined by 1.5 SD above the mean). Exercised participants had higher maximum HR relative to non-exercised participants [*t*(1,68) = 39.58, *p* < 0.001].

### Exercise intensity

Participants’ maximal and submaximal watt data are presented in Table 1. Participants were working on average at 82% of their maximal workload (as defined by submax W/max W × 100) for at least 10 min of the exercise intervention.

### Fear conditioning and extinction

**Fig. 1:**
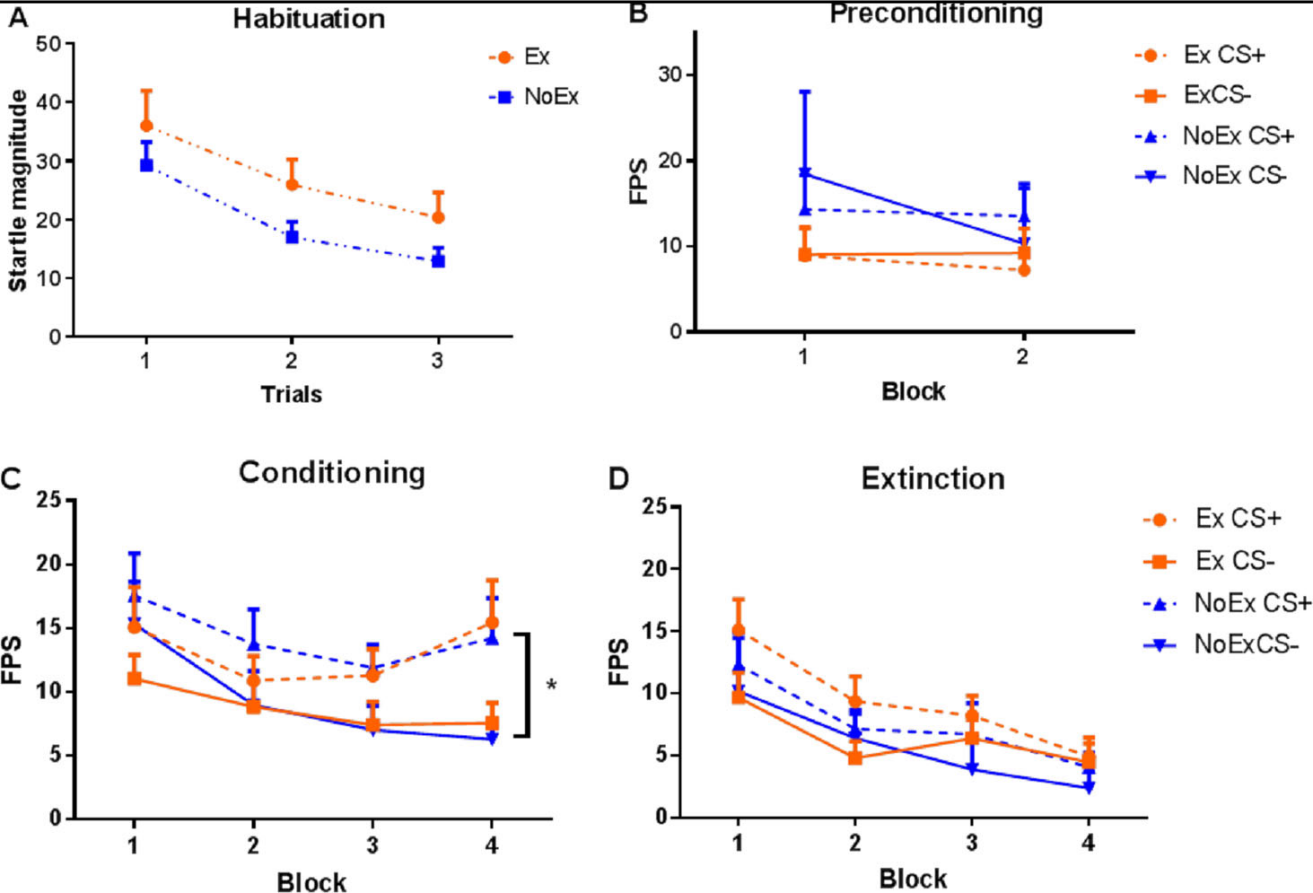
Startle magnitude to CS+ and CS− trials during all phases in session 1. Error bars represent standard error of the mean

**Fig. 2:**
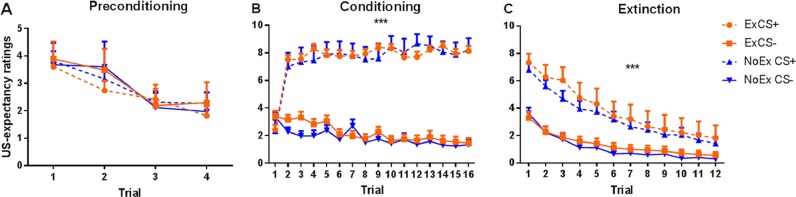
US-expectancy ratings for CS+ and CS− trials during preconditioning, conditioning and extinction. Ex exercise condition, NoEx no exercise condition. Error bars represent standard error of the mean

Startle magnitude and US-expectancy rating data for session 1 are presented in Figs. 1 and 2, respectively.

### Baseline startle magnitude and shock level

The level of shock chosen by participants did not significantly differ by Experimental Condition (*t*(1,51.02) = −1.42, *p* = 0.162). A mixed ANOVA revealed that startle magnitude habituated across blocks (*F*(1.46,99.06) = 33.21, *p* < 0.0001), and this effect did not significantly differ between exercise groups.

### Preconditioning

A mixed ANOVA revealed that fear potentiated startle did not differ by Conditioned Type or Experimental Condition, suggesting no group differences in response to the to-be conditioned CS+ or CS−.

### Fear conditioning

As expected, overall fear potentiated startle to the CS+ was greater relative to the CS (*F*(1,68) = 14.45, *p* < 0.0001), and a significant Assessment Block × Conditioned Type (*F*(3,166.79) = 3.10, *p* = 0.038) interaction. There was no between-group differences.

### Fear extinction

All participants displayed a significant extinction of fear potentiated startle to the previously reinforced CS+ [*F*(1,68) = 16.63, *p* < 0.001] and Assessment Block [*F*(3,151.90) = 19.12, *p* < 0.0001], with no effects for Experimental Condition.

### US-expectancy ratings

During preconditioning, a linear main effect of trial number was found for expectancy ratings (*F*(2.09, 142.23) = 19.46, *p* < 0.001), suggesting that shock expectancy ratings reduced across number of CS presentations, suggesting no discrimination between CSs. During conditioning, there was a main effect of Conditioned Type, (*F*(1,68) = 507.78, *p* < 0.0001), and a significant Trial Number × Conditioned Type (*F*(7.93,542.91) = 37.42, *p* < 0.0001) interaction. Overall, participants displayed clear discrimination between the CS+ and the CS− across trials of the conditioning phase. During extinction, participants displayed a significant reduction in their expectancy ratings to the previously reinforced CS+, reflected by a significant main effect of Conditioned Type (*F*(1,68) = 94.11, *p* < 0.0001), and Trial Number × Conditioned Type (*F*(4.38,297.77) = 17.07, *p* < 0.0001) interaction.

### Recall

**Fig. 3:**
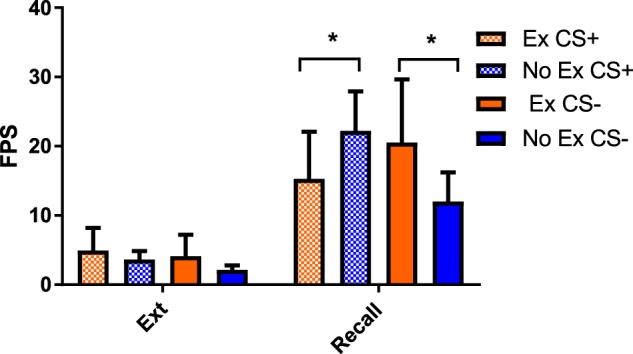
Memory recall. Ex exercise condition, NoEx no exercise condition, Ext extinction. Error bars represent standard error of the mean

A 2 (Experimental Condition) × 2 (Conditioned Type: CS+, CS−) × 2 (phase: final extinction block, initial recall block) repeated measures ANOVA of extinction recall revealed a significant Experimental Condition by Trial Type [*F*(1,65) = 6.19, *p* = 0.015] and a significant Experimental Condition × Conditioned Type × Phase Interaction [*F*(1,65) = 5.91, *p* = 0.018]. Follow-up 2 (Experimental Condition) × 2 (Conditioned Type: CS+, CS−) mixed ANOVAs were conducted for each phase separately. Exercised relative to control participants displayed a significantly lower return of fear potentiated startle response to the CS+ when presented with a retention test 24 h after extinction training. Contrastingly, exercised participants displayed a higher return of fear potentiated startle response to the CS− relative to non-exercised participants [*F*(1,65) = 6.45, *p* = 0.014]. Fear recovery as indexed in fear potentiated startle scores to the CS+ and CS− are presented in Fig. 3.

### US-expectancy ratings at Recall

Contingency awareness on Day 2 analyses indicated that threat expectancy to the CS+ recovered upon exposure to the extinction context overall [Conditioned Type × Phase Interaction, *F*(1,68) = 9.87, *p* = 0.002], however this did not differ by exercise condition.

### Role of BDNF genotype

There were no significant differences between allelic groups in age, gender, DASS21 scores, LTEQ scores, or maximal or submaximal watt levels (see Supplementary Materials, Table S1).

### Fear conditioning and extinction as a function of BDNF Val66Met

Separate 2 (BDNF genotype) × 2 (Condition) × 2 (Conditioned Type) mixed ANOVAs controlling for gender were conducted to assess the impact of BDNF genotype on exercise-induced fear conditioning and extinction. Habituation of startle magnitude did not significantly differ by genotype (non-significant Genotype × Assessment Block, *F*(1.45, 92.60) = 0.021, *p* = 0.947), in the habituation phase. In the preconditioning phase, fear potentiated startle to the to-be conditioned CS+ or CS− were not detected in the preconditioning phase as a function of genotype. Fear potentiated startle to the CS+ relative to the CS− did not differ by BDNF genotype in the conditioning nor extinction phases.

### Moderating effect of BDNF genotype on fear recovery

**Fig. 4:**
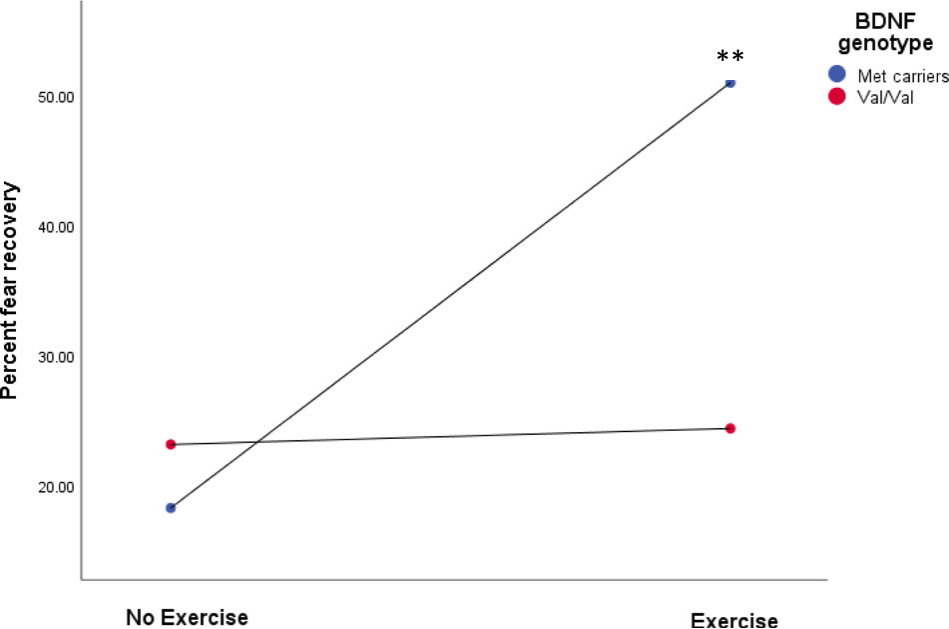
Moderating effect of BDNF Val66Met polymorphism on condition (Exercise, No Exercise) and Fear extinction retention. Percent fear recovery during the recall phase defined as averaged FPS to the CS+ divided by the largest FPS to the CS+ during conditioning

The moderation analysis of BDNF Val66Met genotype indicated a significant association was found between exercise condition and fear recovery (*t*(69) = 2.45, *p* = 0.017; Table 2), such that the exercise condition displayed higher fear recovery relative to the control condition. Further, a significant interaction between exercise condition and BDNF genotype (*t*(69) = −2.23, *p* = 0.029; Table 2) on total fear recovery was found, which added 5.7% of independent variance to the model (*R*^2^ = 0.0571, *p* = 0.0290; Table 2). Simple slope analyses revealed no significant association between exercise condition and fear recovery for Val/Val homozygotes, however the association significantly differed for Met allele carriers (*β* = 0.32, 95% CI[0.09, 0.57], *t* = 2.77, SE = 0.12, *p* = 0.007). This significant negative relationship between condition and fear recovery in Met allele carriers revealed the manner in which exercise was related to impaired extinction retention (indexed by higher FPS to the CS+; see Fig. 4).

**Table 2:** Moderation analysis of BDNF genotype on the relationship between exercise condition and extinction retention as measured by fear potentiated startle

	*B*	SE	*T*	*p*	95% CI	
					Lower	Upper
*Predictor*						
Constant	0.170	0.106	1.606	0.113	−0.042	0.382
Condition	0.176	0.072	2.449	0.017*	0.033	0.310
BDNF	−0.111	0.072	−1.540	0.129	−0.254	0.033
Condition × BDNF	−0.314	0.141	−2.234	0.029*	−0.595	−0.033
Gender	0.088	0.074	1.178	0.243	−0.608	0.236
**Conditional effect of condition on extinction retention at values of the BDNF genotype**						
*BDNF genotype*						
Met	0.326	0.118	2.773	0.007*	0.091	0.562
Val/Val	0.012	0.078	0.159	0.874	−0.143	0.167

*p < .05

## Discussion

Findings of the current study indicated that a bout of intense exercise performed following extinction training reduced conditioned fear on a 24 h delayed retention test. Additionally, an interaction between BDNF genotype and exercise condition significantly predicted fear recovery such that Met allele carriers, but not Val homozygotes, displayed impaired retention of extinction training. This is the first study to extend recent animal research demonstrating that a single bout of wheel running enhances extinction memory consolidation^19,22^, and provides initial evidence that this influence may be moderated by BDNF genotype.

The mechanisms for these findings are speculative. First, it is possible that intense physical exercise following extinction training facilitated activation of biological mechanisms responsible for augmented extinction memory-related plasticity^8,13^. Participants exercised at 82% of their maximal capacity for at least 10 min, and there is research to suggest that this level of intensity facilitates the release of BDNF and stress hormones, including glucocorticoids and noradrenaline^30,52–56^. While this is a likely possibility, further studies must replicate this finding and identify the relevance of these purported biological mechanisms in driving exercise-induced extinction memory retention. Second, it is possible that acute physical exercise acted as a cognitive enhancer in dampening spontaneous recovery within the context of limited exposure to corrective information. The nature of maximal graded cycling may have also functioned as an excitatory stimulus^57,58^, and when implemented in close proximity to extinction training may have served to boost the retention of corrective information via a novelty facilitated mechanism^59,60^. This possibility arises from rodent evidence that exploration of a novel open-field following weak extinction significantly reduced freezing behaviour on a delayed retention test^61^.

This is the first study to evidence a genetic moderation of exercise-induced extinction memory retention. It is possible that exercise magnified genotype dependant differences, such that Met allele carriers were compromised by dysregulated BDNF expression^10^. Met allele carriers may have experienced heightened exercise-induced stress in the absence of regulated BDNF expression, and this in turn interfered with subsequent consolidation of extinction training. This possibility is consistent with evidence that exercise heightens glucocorticoid production^62^, and Met allele carriers display increased cortisol production relative to Val homozygotes^63^. This proposition must be understood in the context of glucocorticoid-enhanced consolidation of inhibitory learning^64,65^ may rely upon regulated BDNF signalling and expression^15^. It is worth mentioning, however, that Met carriers have also been found to display deficient cortisol production in response to stressor tasks^50,51^, albeit in male participants only. Given divergent gender differences in the manner in which BDNF polymorphism impacts reactivity to stress^49,51^, and exercise can be considered a physical stressor, it would be important for future studies to disentangle the extent to which this gender × stress interaction impacts upon subsequent exercise-induced BDNF expression and related extinction retention. This may be especially relevant with recent rodent research evidencing exercise-induced enhancement of extinction retention that is limited to male rodents^19^.

It is worth juxtaposing the current genotype dependant findings with evidence that exercise may compensate for Val66Met deficiencies. Specifically, a self-reported history of physical activity may overcome the deleterious effect of the Met allele on memory performance^66^. Having stated this, it is likely that a single bout of intense physical exercise may not sufficiently increase BDNF secretion to benefit memory consolidation in Met allele carriers. To this end, it has been hypothesised that in active Met carriers, altered mature BDNF inefficiently binds to its receptor TrkB; whereas the precursor form of BDNF (i.e. proBDNF) remains unaltered and binds to its receptor p75, which however results in apoptosis^67,68^. To this end, increased proBDNF levels following acute physical exercise in Met allele carriers may result in increased apoptotic changes in the brain^68^ including impairments in regions responsible for modulating extinction retention^67,69^. In contrast, regular physical activity may counteract volumetric reductions of the hippocampus in rodents^70,71^ and augment the dose of BDNF secretion following acute bouts of physical activity in humans^72^. As such, it remains to be investigated whether an acute bout of exercise within the context of a potent history of exercise (characterised by regular aerobic physical activity) may compensate for deficient intracellular signalling in Met allele carriers, such that this in turn leads to improved benefits from exercise-induced memory consolidation.

This study highlights that Met allele carriers of the BDNF Val66Met polymorphism may pose as a boundary factor in terms of acute exercise-induced benefits on extinction memory retention. While premature to conclude, this finding may help explain inconsistent findings in rodent research whereby exercise is associated with both an increased^22^, and a null effect on^73^ retention of extinction training. Notably, in humans exercise has been found to both augment^74^ and yield no benefit^73^ on outcomes for exposure therapy. These divergent findings may be attributed to differences in sex distribution, timing of exercise (i.e. before vs. after learning) or variants of the BDNF polymorphism. In this context, it is worth noting that these previous studies have varied in their ethnic composition, with one study comprising mainly Caucasians^74^ and the other primarily Asians^73^. This distinction is relevant as the Met allele approximates a 20–30% occurrence in Caucasian populations^75,76^ compared to nearly 50% occurrence in Asian populations^77–79^. Given the evidence that Met carriers may be less responsive to the benefits of physical exercise on cognition^80,81^, the extent to which improvements in exercise-induced consolidation of exposure therapy may be purportedly limited to BDNF Val homozygous carriers in primarily Caucasian samples^74^ requires some consideration.

We note a number of methodological limitations. First, the lack of exercise-induced memory benefits on US expectancy ratings may be attributed to ambiguous wording regarding threat expectancy ratings in that participants were asked to rate how likely a shock would follow a given CS with no specification about context. Second, the current study controlled for gender differences in genotype dependant analyses, however future replications of the study need to be adequately powered to account for potential divergent gender responses to BDNF polymorphism modulated extinction retention. Third, the use of non-clinical participants precludes inferences regarding applicability to anxious populations. Fourth, we acknowledge that exercise may also enhance consolidation of fear conditioning, in line with previous rodent findings^2^. Future research may improve on the current design such that modulation of distinct memories (i.e. conditioning, extinction) may be sufficiently investigated (e.g. delayed extinction protocol). It is also worth considering the prolonged effect of exercise on extinction retention and related spontaneous recovery, and to this end, future studies may assess this via additional delayed extinction retention tests (e.g. 7 days later).

Overall, the current study provides initial evidence that intense physical activity can augment consolidation of extinction in humans. The study additionally sheds light upon mechanisms underlying exercise-induced extinction retention whereby a genetic predisposition involving the BDNF Val66Met polymorphism may pose as a boundary condition for the extent of these memory inducements. These findings are promising and point to potential opportunities for augmenting exposure therapies for anxiety disorders.

## Supplementary information

Supplementary information.
