# Supplementary material for: Acute exercise-induced enhancement of fear inhibition is moderated by BDNF Val66Met polymorphism
Source: Transl Psychiatry. 2019 Apr 9;9:131. doi: 10.1038/s41398-019-0464-z (PMC6456490; doi:10.1038/s41398-019-0464-z)
Supplement: Supplementary file 1 — Supplementary information. [file 41398_2019_464_MOESM1_ESM.docx]

***Supplemental Information***

**Supplemental Methods and Materials**

*Power Analysis*

To determine the required sample size, we conducted an a priori power analysis derived from a previous study of fear acquisition that assessed the impact of environmental manipulation on return of fear after the extinction phase (Hornstein, Fanselow, & Eisenberger, 2016). Using the parameters of that prior study (Cohen’s *d* = 0.68), the power analysis indicated that to identify an effect of our manipulation on subsequent fear recall, we would require at least 35 participants in each condition (95% power, α = .05, two-tailed). We oversampled to achieve a total sample of at least 70 participants allowing for exclusions due to technical issues, drop-outs, and failing to learn contingencies.

*Godin-Shephard Leisure-Time Exercise Questionnaire (LTEQ).* The Leisure-Time Exercise Questionnaire [LTEQ;(1)] was used to index the frequency of light-intensity, moderate-intensity, and vigorous-intensity leisure-time physical activity undertaken on a weekly basis. This questionnaire has good construct validity and strong test re-test reliability (1). The LTEQ was modified by asking participants to indicate how much time (in minutes) is spent on strenuous, moderate and light activity for more than 15 minutes in a given 7-day period, such that the duration of engagement in each type of activity could be assessed, and factored into when estimating weekly metabolic equivalent (METS). A modified formula was utilised to calculate METS: [(total METS = minutes of strenuous exercise / 15) × 9] + [(total minutes of moderate exercise / 15) × 5] + [(total minutes of light exercise / 15) × 3] (2).

*Heart rate measurement.* A Garmin FR70 heart rate watch and chest belt consisting of 2 smart fabric sensors measured cardiac activity, and this index participants’ heart rate. Heart rate data was sampled at a rate of 2.4 GHz, and wirelessly transmitted to the Garmin Connect Training centre.

*Perceived Exertion.* The Borg Rating of Perceived Exertion scale [RPE; (3)] indexed subjective ratings of exercise intensity such that participants’ effort on the assigned exercise tasks could be assessed. This measure takes into account individual fitness level by requiring participants to match how hard they feel they are working on a 15-point scale (6 = No exertion at all, 20 = Maximal exertion). Moderate activity is classified as 11–14, and vigorous activity as 15 or above. This measure provides an approximation of heart rate by multiplying a given rating by 10 (e.g. if an individual’s rating of perceived exertion (RPE) is 16, then 16 × 10 = 160, suggesting that their heart rate should approximate 160 beats per minute (3, 4).

*Exercise workload.* Participants’ workload on the cycle ergometer was determined using the following formula: Work (kgm) = force (kg) × distance (meters/min) where force relates to the frictional resistance applied on the bike as measured in Kg, and distance refers the cadence (rev/min) multiplied by the a constant of 6 (i.e., whereby 6 m per revolution of the flywheel applies to the Monark ergometer). For example, if an individual is working at a frictional resistance of 2.5 kg at 65 RPM, then the work level (kgm) engaged would be defined as 2.5 kg × 65 RPM × 6m/rev = 975. Work level is then expressed in watts approximately equivalent to 6kgm/min, thus the individual in the above example would be exercising at a 162.5 W (975kgm/min divided by 6). Accordingly, maximal- and submaximal watt was calculated using the highest- and lowest level of resistance endured by participants at a minimum cadence of 65 rpm respectively.

*Conditioning Procedure.* To reduce variation in stress hormones, participants were required to refrain from eating 1-hour prior, taking caffeine, alcohol or nicotine 3 hours prior to, and exercising the day before, both experimental sessions. The CSs were yellow and purple square geometric shapes (15.2cm), which were placed within a grey or white color background (contexts; CXs), where one served as the “conditioning context” and the other as the “extinction context”. The selection of the CS+ and CS- colors, and the CX+ and CX- backgrounds were randomly determined and counterbalanced across participants. The unconditioned stimulus (US) was a 0.5sec mild electric shock produced by a constant current stimulator that was delivered via a stainless steel bipolar electrode (ADInstruments) that was Velcro strapped to the inside of the wrist of the participant. Intensity of the US was determined with a threshold test (range, 3- 19.8mA), until participants indicated that the shock was “highly annoying but not painful” (5). Contexts and CSs were displayed on a computer screen (28.5 x 21.5 cm monitor) positioned approximately 61cm in front of the participant, at the level of the face. Each trial began with a 0.5sec fixation cross followed by a 10.5sec context presentation: 3.5sec alone followed by 7.5sec or 7.0sec in combination with the CS+ or CS- respectively (see Figure 1a). The mean inter-trial interval (ITI) was 20sec (range: 16sec – 24sec), and was determined from the offset of the CS to the onset of the context. The acoustic startle stimulus (i.e., startle probe) was a 40-ms duration 100dB burst of white noise with near instantaneous rise-time, and this was presented through binaural headphones against a background noise of 65dB.

Figure 1b details specific timings of CS trials. On CS+ trials, the shape was presented for a total of 7.5sec, with the 40ms startle probe presented 6s after the CS onset, followed 1sec later by the .5sec electric shock that co-terminated with the CS presentation. On CS- trials, the shape was presented for a total of 7.0sec, with the startle probe occurring at 6sec after the CS onset. On startle alone trials the 40-ms startle probe was presented alone during every second ITI. The startle alone trials were not presented within 8sec of the offset of a CS to the onset of the following CS in order to prevent possible shock sensitisation on startle reactivity (7). CSs were presented in a pseudo random order, such that no CS appeared more than three consecutive times. All phases on Day 1 (i.e., preconditioning, conditioning, and extinction), and Day 2 (i.e., recall) began with the delivery of four startle stimuli to further habituate startle (data not presented).

*Differential fear conditioning paradigm apparatus.* Two computers ran LabChart 8 software (ADInstruments: Sydney, Australia) and Presentation 18.2 software (Neurobehavioral Systems, Inc: San Franciso), both of which were used to control data acquisition and stimulus events, respectively. The eye blink component of the startle response was measured by recording activity of the orbicularis oculi electromyogram (EMG) with two 4mm Ag-AgCl electrodes filled with electrolyte gel. One electrode was placed 1 cm below the pupil of the left eye and the other 1cm lateral [centre to centre; (6)]. The ground electrode was placed at the back of the neck. Impedance level was kept below 5 KΩ. Raw EMG activity was amplified and digitized at 1000Hz using the AD Instruments Dual Bio Amp (FE135). The signal was then filtered (band-pass = 28-500 Hz), rectified and smoothed using a 25msec moving averages window (LabChart, AD Instruments).

*US expectancy ratings*. Online US expectancy ratings were assessed for each trial during the preconditioning, conditioning, extinction, and recall phases. Participants were asked to rate the extent to which they expect to receive a shock on a 10-point scale (0 = *do not expect to receive a shock*, 10 = *definitely expect to receive a shock*) each time a CS was presented on screen. All ratings were presented on a response keypad.

*Instructions to participants.* On Day 1, the shock electrode was connected following the habituation phase and prior to the preconditioning phase. At the start of the preconditioning phase, participants were instructed that once the experiment began, on each trial they “may or may not be shocked”, and that if they paid attention to each trial they could potentially predict the administration of shocks. Such an instruction was utilised to maximise awareness during the experiment (8). At the beginning of the extinction phase, participants were reminded that they “may or may not get shocked”. On Day 2, the shock electrode was once again attached after the startle habituation phase and remained attached until the end of the session, although no US was administered. Participants were instructed that they would complete similar tasks to the previous day. Once again they were told that they “may or may not get shocked” prior to each phase of the experiment.

***Primary data extraction***

Peak magnitude of the blink reflex was calculated as the maximum of the smoothed response within 21-120ms of the startle stimulus onset relative to a baseline value. This baseline value was calculated by taking average EMG activity in the 20ms preceding the onset of the startle probe. Trials that indicated excessive EMG activity during the first 20ms were omitted. Outliers in EMG data defined as 3 SD above the mean were replaced with the mean plus three times the standard deviation. SPSS statistical package (v.25) was used to conduct data analyses. In order to assess potentiation to a CS+ or CS- relative to a startle alone (SA) trial, a difference score was calculated using the following formula: Fear potentiated startle (FPS) = [Peak startle magnitude to startle probe in the presence of CS+ or CS-] – [Peak startle magnitude to the startle probe alone (SA)] (9).

*
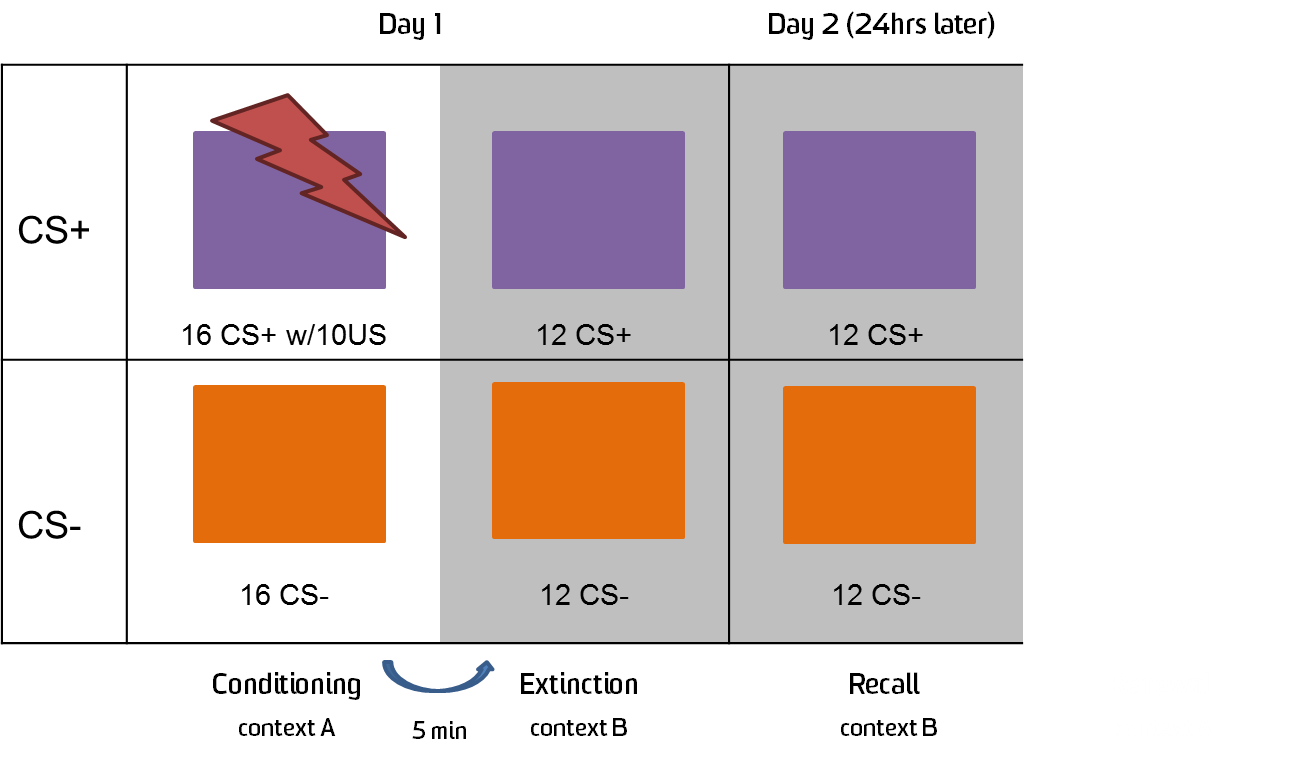
*

*Figure S1a.* Experimental protocol


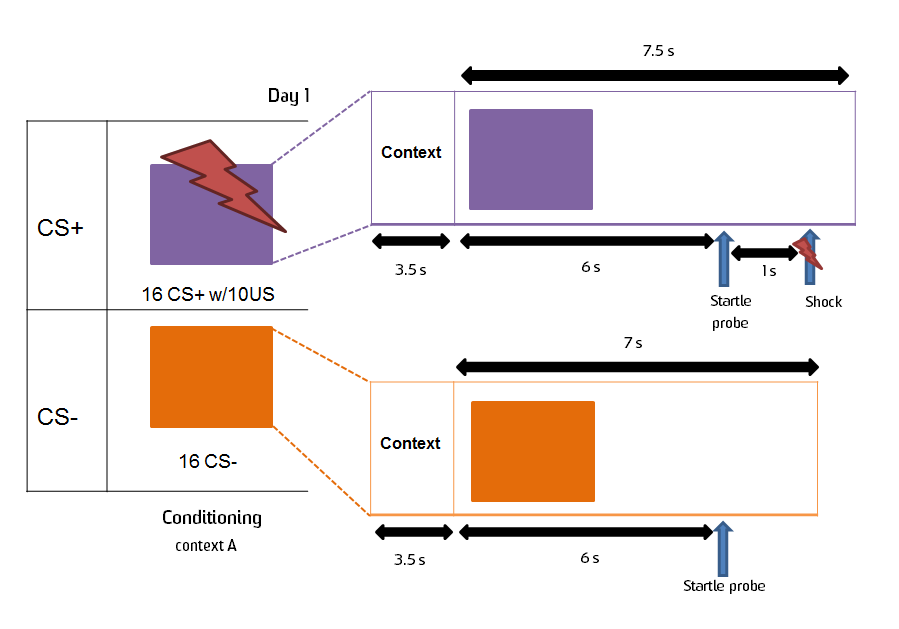


*Figure S1b.* Timing of CS+ and CS- trials

Table S1

*Participant characteristics by BDNF genotype*

|  | Val/Met or Met/Met (n=36) | Val/Val (n=33) |
| --- | --- | --- |
| Age  Gender | 21.00 (3.98)  19M, 14F | 20.27 (2.05)  20M, 16F |
| DASS-depression | 2.31 (2.51) | 2.82 (2.40) |
| DASS-anxiety | 2.61 (1.93) | 3.27 (2.76) |
| DASS-stress | 3.67 (2.47) | 4.85 (3.61) |
| Maximal watt | 69.06(75.96) | 91.10 (91.20) |
| Submaximal watt | 57.78 (64.35) | 73.96 (75.68) |
| LTEQ light intensity (minutes) | 50.00 (360.00) | 50.00 (480.00) |
| LTEQ moderate intensity (minutes) | 90.00 (450.00) | 60.00 (900.00) |
| LTEQ strenuous intensity (minutes) | 60.00 (390.00) | 60.0(1080.00) |

*Note:* Standard deviations appear in parentheses. Median and range (in parentheses) for LTEQ subscales have been presented in the table above.

**Supplemental references**

1. Godin Shephard, R. J. G (1997): Godin Leisure-Time Exercise Questionnaire. *Med Sci Sport Exerc*. 29: 36–38.

2. Andrykowski MA, Beacham AO, Jacobsen PB (2007): Prospective, longitudinal study of leisure-time exercise in women with early-stage breast cancer. *Cancer Epidemiol Biomarkers Prev*, 2007/03/21. 16: 430–438.

3. Borg GA V (1982): Psychophysical bases of perceived exertion. *Med Sci Sport Exerc*. 14: 377–381.

4. Johnson JH, Prins A (1991): Prediction of maximal heart rate during a submaximal work test. *J Sport Med Phys Fit*, 1991/03/01. 31: 44–47.

5. Orr SP, Metzger LJ, Lasko NB, Macklin ML, Peri T, Pitman RK (2000): De novo conditioning in trauma-exposed individuals with and without posttraumatic stress disorder. *J Abnorm Psychol*, 2000/07/15. 109: 290–298.

6. Blumenthal TD, Cuthbert BN, Filion DL, Hackley S, Lipp O V, van Boxtel A (2005): Committee report: Guidelines for human startle eyeblink electromyographic studies. *Psychophysiology*, 2005/02/22. 42: 1–15.

7. Davis M (1989): Neural systems involved in fear-potentiated startle. *Ann N Y Acad Sci*, 1989/01/01. 563: 165–183.

8. Milad MR, Orr SP, Pitman RK, Rauch SL (2005): Context modulation of memory for fear extinction in humans. *Psychophysiology*, 2005/07/13. 42: 456–464.

9. Norrholm SD, Anderson KM, Olin IW, Jovanovic T, Kwon C, Warren VT, *et al.* (2011): Versatility of fear-potentiated startle paradigms for assessing human conditioned fear extinction and return of fear. *Front Behav Neurosci*, 2011/11/30. 5: 77.
